# Quality of life of people living with HIV/AIDS: temporal, socio-demographic and perceived health relationship**^1^**

**DOI:** 10.1590/1518-8345.1258.2874

**Published:** 2017-04-20

**Authors:** Rodrigo Leite Hipolito, Denize Cristina de Oliveira, Tadeu Lessa da Costa, Sergio Corrêa Marques, Eliane Ramos Pereira, Antonio Marcos Tosoli Gomes

**Affiliations:** 2 PhD, Adjunct Professor, Escola de Enfermagem Aurora de Afonso Costa, Universidade Federal Fluminense, Niterói, RJ, Brazil.; 3 PhD, Full Professor, Faculdade de Enfermagem, Universidade do Estado do Rio de Janeiro, Rio de Janeiro, RJ, Brazil.; 4 PhD, Adjunct Professor, Faculdade de Enfermagem, Universidade Federal do Rio de Janeiro, Macaé, RJ, Brazil.; 5 PhD, Adjunct Professor, Faculdade de Enfermagem, Universidade do Estado do Rio de Janeiro, Rio de Janeiro, RJ, Brazil.; 6 PhD, Associate Professor, Escola de Enfermagem Aurora de Afonso Costa, Universidade Federal Fluminense, Niterói, RJ, Brazil.

**Keywords:** Quality of Life, Human Immunodeficiency Virus, Acquired Immunodeficiency Syndrome, Nursing

## Abstract

**Objective:**

to analyze the quality of life of people living with HIV/AIDS and its relationship with sociodemographic variables, health satisfaction and time since diagnosis.

**Method:**

quantitative, cross-sectional study with a sample of 100 HIV positive people monitored in a specialized service in southeastern Brazil. Sociodemographic and health forms were applied, followed by the WHOQOL-HIV BREF, a short form instrument validated to evaluate the quality of life. Descriptive and inferential statistical analysis was performed.

**Results:**

the perception of quality of life was intermediate in all quality of life domains. A relationship was identified between greater satisfaction with health and better quality of life, as well as statistically significant differences among the dimensions of quality of life according to gender, employment status, family income, personal income, religious beliefs and time since diagnosis.

**Conclusions:**

the time since the diagnosis of HIV infection enables reconfigurations in the perception of quality of life, while spirituality and social relationships can assist in coping with living with this disease.

## Introduction 

The use and access to antiretroviral therapy (ART), monitoring by health services and the related outcomes, highlight a route in the quest for improving the QoL of people living with HIV/AIDS (PLWHA)^1^. However, finding a definitive solution to the problem is still a challenge faced by scientists from all disciplines, in relation to this area of ​​study. Together with the search for a cure, investments in the prevention of transmission of the human immunodeficiency virus (HIV) are equally necessary^2^. The PWHA that face difficulties in treatment, due to poor quality or lack of access, can manifest adherence-related problems, potentially increasing the risk of disease transmission and reducing the QoL^3^.

It is estimated that, at the end of 2014, approximately 781,000 people were living with HIV/AIDS in Brazil, representing a HIV prevalence rate of 0.39%. Of these PLWHA, 83% (649,000) had been diagnosed. Approximately 80% were linked to the health service at some point after diagnosis. However, only 66% remained in these services^2^. Clinical monitoring of PLWHA is currently one of the most important tools for following the progress and efforts needed to improve their care. More than half (52%) of PLWHA were undergoing ART (405,000), and 46% (356,000) of them presented viral suppression at least six months after the start of the ART^2^. 

Given that ART increases the survival of PLWHA, it is considered that healthcare becomes very important for these individuals, who require singular care to maintain the QoL^4^. Relating the concept of QoL with the current context of rapid and intense social change is necessary to overcome the demands inherent in society and to understand QoL as a human way of perceiving one's own existence, from objective and subjective spheres^5^.

For the assessment of QoL, the World Health Organization (WHO) developed a universal instrument (WHOQOL-100), as well as a short form, the WHOQOL-BREF, which enables a more rapid application. Both were proposed by the QoL assessment group of the WHO, The WHOQOL Group^6^. This instrument provides an assessment that is not only based only on the clinical parameters of HIV infection, also considering the biopsychosocial dimensions, notably central in the lives of these people^6^. 

The WHO defines quality of life, as "an individual's perception of their position in life in the context of the culture and value systems in which they live and in relation to their goals, expectations, standards and concerns"^6^. Studies show how the social conditions, personal and cultural values ​​and clinical factors influence the QoL^7^ ^-^ ^9^.

In order to enable an approach to the QoL phenomenon of PLWHA and considering that the social elements have an impact on this process, this study was proposed to analyze the quality of life of people with HIV/AIDS and its relationship with sociodemographic variables, health satisfaction and time since diagnosis. The study is also justified considering that, in addition to socio-demographic and health variables, time since diagnosis and living with the infection play an important role in how PLWHA perceive their QoL, being measures useful for nursing care.

## Method

This cross-sectional, quantitative study was developed in a STD/AIDS and Viral Hepatitis Program of a coastal municipality in the state of Rio de Janeiro, Brazil. The program had 352 people, registered in January 2014, diagnosed as HIV positive and with AIDS, receiving multidisciplinary care, having been referred or arrived spontaneously.

To achieve the aims of the study, a convenience sampling of 100 PLWHA was defined, equivalent to 28.4% of the users registered in the service during the data collection period. The criterion for definition of candidates for inclusion in the study sample was their presence on the days of medical care in the unit. 

The subjects were invited to participate in the study while awaiting consultation with an infectious disease specialist in the waiting room, taking into consideration the time since diagnosis, which was revealed soon after acceptance to participate in the study. The collection was carried out during the period between May 2013 and January 2014 on Mondays and Thursdays, the days of medical consultations, favoring the encounter with the largest number of registered clients. Eight people refused to participate and 11 instruments were discarded due to inadequate completion. This did not impair the sample, as new participants were included until it was complete.

The study included PLWHA aged over 18 years, who were enrolled in the previously mentioned STD/AIDS and Viral Hepatitis Program and had clinical and cognitive conditions compatible with the study issues, which were identified by questioning the PLWHA. Furthermore, they needed to feel that they were in physical condition compatible with the demands of the study and present proper understanding of the questions of the study instrument. Those who did not meet the inclusion criteria and those that received ART through the program, however, were monitored in a private service were excluded. 

Regarding the data collection, two instruments were applied with the participants in private. This collection took place initially with the application of a form consisting of 20 closed questions, including identification, socioeconomic and health data. Next, the specific instrument developed by the WHO, the WHOQOL-HIV BREF was used for the assessment of QoL^10^. This form is designed to be self-administered, therefore, one of the researchers remained next to the respondent awaiting its completion and provided clarifications where necessary.

The WHOQOL-HIV BREF produces six domain scores, these being: physical, psychological, level of independence, social relationships, environment and spirituality/religion/personal beliefs. These domains contain 29 facets of one item each, with a total of 29 items. Five facets are specific to people living with HIV/AIDS: symptoms, social inclusion, forgiveness and blame, concerns about the future, and death and dying. In addition to the 29 facets, there are two questions that examine the overall QoL: Question 1 that investigates the individual's overall perception of their QoL and Question 2, which evaluates their general health perception, totaling 31 items^11^. 

The questions are individually scored on a Likert type scale, where 1 indicates low negative perceptions and 5 indicates high positive perceptions. However, some facets are not formulated positively, meaning that for these facets, higher scores do not denote better QoL, needing to be reversed or recoded during the data analysis. It should be noted that the questions are organized by response scale, i.e., capacity, frequency, intensity or satisfaction^11^ and the scores range from 4 to 20 points, reflecting the worst and the best QoL, respectively^10^.

The data obtained were organized in Excel spreadsheets and exported to the Statistical Package for the Social Sciences (SPSS) version 17.0 program. For the descriptive statistics, the absolute and relative frequency, mean, standard deviation, coefficient of variation, and minimum and maximum values were used. To characterize the assessment of QoL, the classification from some previous studies on the same population was used^4^ ^,^ ^7^ ^,^ ^12^. In this, a score between 4 and 9.9 was considered a lower position, from 10 to 14.9 intermediate and 15 to 20 high.

The normality between the sampling means was tested, using the Kolmogorov-Smirnov and Shapiro-Wilk tests. As there was no normal distribution between the six domains, non-parametric tests were used^13^, with the Mann-Whitney U Test used for comparing two groups, specifically for gender and use of ART; and the Kruskal-Wallis (KW) test for the other variables. A significance level of p ≤0.05 was adopted in the comparisons. For the variable time since HIV diagnosis, the tests were primarily applied with the variable categorized in months and then categorized in years. This strategy had the intention of expanding the margin of statistical analysis.

The study was approved by the Research Ethics Committee of the State University of Rio de Janeiro (UERJ), No. 073/2012 and Authorization No. 066.3.2012. The participants signed the consent form, and their anonymity was guaranteed.

## Results

Table 1 presents the sociodemographic and health characterization of the PLWHA. The group was composed equally by men and women, by single people (53%), with a mean age of 37.6 years and with 2 or 3 children (35%). The time since diagnosis of less than 4 years (68%), the use of ART (74%) and length of ART use of less than 4 years (52%) were highlighted. The majority of the subjects lived in the same municipality as the treatment (89%), sharing a residence with 3 to 4 people (39%) of the family (82%). They presented low levels of education (48%), having the treatment site as the main source of information about HIV/AIDS (48%). The majority performed a work and employment activity (61%), with a mean household income of $965.00 and mean personal income of $460.00.

**Table 1:** Distribution of people living with HIV/AIDS, according to sociodemographic and health variables. A coastal municipality of Rio de Janeiro, RJ, Brazil, 2013-2014.

Variables	n	%
Gender		
Male	50	50.00
Female	50	50.00
Marital Status		
Single	53	53.00
Married	34	34.00
With partner	7	7.00
Widowed	6	6.00
Age group		
24 or less	16	16.00
25 to 34	26	26.00
35 to 44	29	29.00
45 to 54	19	19.00
55 or more	9	9.00
Did not answer	1	1.00
Number of children		
None	28	28.00
1	23	23.00
2 to 3	35	35.00
4 to 5	12	12.00
More than 5	2	2.00
Time since HIV diagnosis (years)		
4	68	68.00
5 to 8	17	17.00
9 to 12	8	8.00
13 to 16	4	4.00
17	3	3.00
Use of antiretroviral therapy		
Yes	74	74.00
No	26	26.00
Length of antiretroviral therapy use (years)		
4	52	52.00
5 to 8	11	11.00
9 to 12	7	7.00
13 to 16	4	4.00
Resident of the municipality		
Yes	89	89.00
No	11	11.00
No. of people sharing residence		
0	11	11.00
1 - 2	37	37.00
3 - 4	39	39.00
5 - 6	13	13.00
Group of people sharing residence		
Family	82	82.00
Friends	3	3.00
Did not answer	4	4.00
Did not apply	11	11.00
Level of education		
Incomplete elementary	14	14.00
Complete elementary	5	5.00
Incomplete high school	29	29.00
Complete high school	39	39.00
Incomplete higher	6	6.00
Complete higher	5	5.00
Postgraduate degree	2	2.00
Source of information		
Health professionals	48	48.00
Books and magazines	30	30.00
Did not answer	13	13.00
Television	9	9.00
Employment status		
Unemployed	39	39.00
Self-employed	30	30.00
Formal contract	25	25.00
Temporary contract	4	4.00
Public worker	2	2.00
Family income in dollars*		
Less than 300	17	17.00
301 to 600	33	33.00
601 to 900	15	15.00
901 to 1200	8	8.00
1201 to 1500	10	10.00
Above 1500	10	10.00
Did not answer	7	7.00
Personal income in dollars*		
Less than 300	20	20.00
301 to 600	30	30.00
601 to 900	13	13.00
901 to 1200	7	7.00
1201 to 1500	3	3.00
Above 1500	2	2.00
Did not answer	25	25.00
Total	100	100

*Refers to the commercial value of the American dollar in Dec 2013 in Brazil: R$ 2.34.

Table 2 shows the result of the analysis of the first two items of the form, with the aim of verifying a possible relationship between the general perception of QoL and degree of satisfaction with health. Considering that the estimates proposed by the WHOQOL-HIV BREF questionnaire refer to the previous two weeks of life, a similar and crossed distribution could be seen in the data pattern of these variables. This relationship was identified by the higher percentage in the range considered to be a more positive perception of QoL and satisfaction with health, which was "very much" (n = 51, n = 39, respectively).

**Table 2:** Relationship between overall perception of quality of life and satisfaction with health of people living with HIV/AIDS. A coastal municipality of Rio de Janeiro, RJ, Brazil, 2013-2014.

	Degree of satisfaction with health					Total
	None	Very little	More or less	Very much	Extremely	
Quality of life						
Not at all	0	1	0	1	0	2
	0.0%	50.0%	0.0%	50.0%	0.0%	100.0%
A little	3	2	1	0	0	6
	50.0%	33.3%	16.7%	0.0%	0.0%	100.0%
A moderate amount	0	7	12	7	0	26
	0.0%	26.9%	46.2%	26.9%	0.0%	100.0%
Very much	1	6	14	25	5	51
	2.0%	11.8%	27.5%	49.0%	9.8%	100.0%
An extreme amount	1	0	2	6	6	15
	6.7%	0.0%	13.3%	40.0%	40.0%	100.0%
Total	5	16	29	39	11	100
	5.0%	16.0%	29.0%	39.0%	11.0%	100.0%

Table 3 presents the distribution of the parameters relating to the WHOQOL-HIV BREF domains. The perception of intermediate QoL was revealed in all six domains investigated. Regarding the gross mean scores, it was observed that the highest were assigned to the spirituality domain (14.61), which, in addition to personal beliefs, evaluates the perception about the feelings of forgiveness and blame, concerns about the future, and death and dying; and the social relationships domain (14.52), which evaluates the perception of the respondent regarding personal relationships, social support, sexual activity and social inclusion. The lowest means were assigned to the level of independence domain (13.77), which assesses the perception of respondents regarding their mobility, daily living activities, dependence on medication or treatment and ability to work, and the environment domain (13.29), related to the perception of physical security, housing, finance, access to quality services, access to information, leisure, physical environment and transportation.

**Table 3:** Distribution of the scores among people living with HIV/AIDS, according to the WHOQOL-HIV BREF^*^. A coastal municipality of Rio de Janeiro, RJ, Brazil, 2013-2014.

Domain	No. items	Mean	Standard Deviation	Coefficient of Variation	Minimum	Maximum
Spirituality/religion/personal beliefs	4	14.61	3.78	25.89 %	5.00	20.00
Social relationships	4	14.52	3.88	26.74 %	4.00	20.00
Psychological	5	14.00	3.25	23.19 %	4.80	20.00
Physical	4	13.85	3.60	25.99 %	5.00	20.00
Level of independence	4	13.77	3.21	23.31 %	6.00	19.00
Environment	8	13.29	2.34	17.59 %	6.50	19.00

*WHOQOL-HIV BREF: World Health Organization Quality of Life instrument-HIV BREF.

Table 4 presents the results of the comparative statistical analysis between the categories of the temporal and sociodemographic variables, the collation of which, according to the WHOQOL-HIV BREF, showed statistically significant differences with respect to: gender; employment status; family income; personal income; religion/spirituality/personal beliefs and time since diagnosis. The men presented a better perception of QoL in the level of independence domain and the individuals categorized as public workers in the physical and social relationships domains, although the latter statistical analysis had the limitation of being a very small group, with only 2 participants. The per capita income in the range of family earnings between US$1,501 and US$1,800 was represented by the best evaluations in the psychological and environment domains, while those with personal income above $1,501 evaluated better QoL in the spirituality and personal beliefs domain. The Kardecists/Spiritists presented the best evaluation of QoL in the level of independence domain. 

**Table 4:** Distribution of the domains of quality of life evaluation, according to the sociodemographic variables. A coastal municipality of Rio de Janeiro, RJ, Brazil, 2013-2014.

Variables	Physical	Psychological	Level of independence	Social relationships	Environment	Spirituality Religion Beliefs
Gender						
Male			14.51			
Female			13.00			
P-value*			0.0280**^†^**			
Employment status						
Formal contract	15.66			16.80		
Public worker	17.20			17.50		
Temporary contract	13.53			14.07		
Self-employed	13.12			13.40		
Unemployed	12.60			13.50		
P-value*	0.022**^‡^**			0.005**^‡^**		
Family income**^§^**						
Less than $300.00		14.71			13.20	
From $301 to 600		13.28			12.37	
From $601 to 900		15.04			13.83	
From $901 to 1200		16.40			15.31	
From $1201 to 1500		13.28			13.61	
From $1501 to 1800		16.53			16.00	
Above $1800		13.82			14.33	
P-value*		0.032**^‡^**			0.011**^‡^**	
Personal income**^§^**						
Less than $300.00						15.22
From $301 to 600						13.80
From $601 to 900						13.69
From $901 to 1200						18.85
From $1201 to 1500						10.33
Above $1500						19.00
P-value*						0.012**^‡^**
Religion/Spirituality						
Catholic			14.64			
Evangelical			13.11			
Kardecist/Spiritist			16.75			
Umbanda			13.50			
Candomblé			9.75			
Without religion			14.25			
P-value*			0.032**^‡^**			
Time since diag. in years						
Less than 4	14.41					
From 5 to 8	11.29					
From 9 to 12	14.12					
From 13 to 16	13.75					
More than 17	15.00					
P-value*	0.052**^‡^**					

*P-value ≤0.05; †Mann-Whitney test; ‡Kruskal-Wallis test; §Refers to the commercial value of the American dollar in Dec 2013 in Brazil: R$ 2.34.

According to the tests performed it can be verified that, regarding the time since HIV diagnosis categorized in months, the QoL among the groups, in the physical, psychological, level of independence, social relationships, environment and spirituality domains showed no significant differences in the statistical associations. However, a level of significance was evidenced (p 0.052), with higher QoL evaluation scores in the physical domain, in the subgroup with over 17 years of living with the diagnosis. Similar values in the scores for the referred domain were observed in the other subgroups, except the group between 5 to 8 years, with the lowest score. 

## Discussion

Considering the comparison with other studies^4^ ^,^ ^12^, the profile of the subjects is in accordance with recent Brazilian and global data, since the epidemic currently presents a concentration in more vulnerable population subgroups^2^ ^,^ ^14^. The recent Brazilian data show that AIDS is far from being controlled and presents its worst indicators in the more than thirty years of the disease. Since 2011, the barrier of 40,000 new cases per year has been exceeded, with no signs that this will reduce again in a short period of time. A new generation, born after the mid-1990s, has also begun to present higher incidence rates than those recorded among people that began their sexual life after the epidemic started. This epidemiological profile, in a certain way, presents similar characteristics to those seen in the early 1980s, when the first victims of the disease began to be seen, presenting a focus strongly concentrated in specific social segments^15^ ^-^ ^17^.

The diagnosis and treatment of infected people would have the potential to eliminate the occurrence of new infections, which has encouraged the United Nations to invite countries to implement ambitious programs by 2020 to diagnose 90% of the people with HIV, treating 90% of them with antiretroviral drugs, leading to 90% of those treated having undetectable viral loads. It is called the 90-90-90 target that, according to the United Nations, could lead to the end of the epidemic in the world by 2030^1^ ^-^ ^2^ ^,^ ^14^ ^,^ ^16^.

Since the epidemic began, a network of care for people infected has been deployed in the country, based on the principles of integrality and interdisciplinarity, with quality evaluations showing relatively satisfactory structures and work processes for significant numbers of the health units^7^. In recent years, however, part of this network has been penalized, due to the underfunding of the Brazilian National Health System (SUS) and the weakening of the response to AIDS in the country^16^. Given these facts, the analysis of the quality of life of PLWHA in Brazil becomes increasingly important, as the evaluation indicators of the responses to public policies have lead to the effectiveness of integral and holistic care practices aimed at these groups being questioned. 

The results of the overall perception of QoL and satisfaction with the health of PLWHA are similar to those found in another study carried out in a municipality of the northern region of the state of Rio de Janeiro^12^. This showed that the higher the degree of satisfaction with health, the better the QoL. This fact leads to the question that. even being HIV positive. they could consider, from their forms of knowledge elaborated, personal values ​​and beliefs, a good assessment of QoL and a high degree of satisfaction with health, which could contribute to the construction of a reality common to this social group. 

The analysis of the gross mean showed that the better assessments of QoL in the group were associated with perception of meaning of life, re-evaluation of opinions about death, the discovery of new relationships with God and support resulting from social relationships, referring to the spiritual, religion and personal beliefs domain. While the lower scores led to the reflection that this relationship may have a connection with the issues surrounding the use of antiretroviral therapy and its impact on life in general (level of independence domain), and the influence of financial issues on the QoL of PLWHA (environment domain), especially considering the range of family income and education level of the participants. These results are similar to those of other studies^12^ ^,^ ^15^ ^,^ ^18^ ^-^ ^19^. 

The better perception of QoL identified in the group of men in the level of independence domain corroborates studies that show differences in the assessment of QoL between the genders, with the group of women generally identified as disadvantaged in the comparison between domains, explained partly by sociocultural issues^12^ ^,^ ^20^. Over the preceding years, the configuration of the way in which people experience the process of HIV/AIDS in their various spheres and its impact on QoL has been discussed, with the gender variable defining differences in the perception of QoL^7^ ^,^ ^10^ ^,^ ^12^ ^,^ ^15^.

The results of a recent study have also contributed to reveal significant differences in the independence, environment and spirituality domains, as well as the general facet of QoL, according to the transmission of the virus category. The group that reported acquisition of HIV through intravenous drug (IVD) use reported worse QoL score than the group of men who reported transmission due to sex with men (MSM) and the group that reported heterosexual transmission. The latter showed significantly worse QoL in the environment domain and in the general facet than the MSM group. The results suggest important differences according to the mode of HIV transmission and reinforce the importance of QoL promotion interventions tailored to the characteristics and needs of different population subgroups^21^.

A small sample of workers in more stable employment situations presented better QoL in the physical and personal relationships domains, compared to those in less stable situations and unemployed. This may suggest that being employed means more than just financial benefits for these people. Some studies have associated being without paid work with poorer QoL in the majority of domains^12^ ^,^ ^20^. It was observed that, the higher the wage earned in the family, the better perception of QoL in the psychological and environment domains. These data show that the QoL in PLWHA is also determined by economic, social and biopsychosocial aspects^18^.

From the analysis in question, greater individual earnings positively affect the perception of QoL in the domain that deals with questions about the future, personal beliefs, feelings of blame and forgiveness, and concerns about death, which indicates a significant relationship between greater economic power and better QoL, with regard to the way in which the group of subjects project their life expectancies. The issues related to access to goods and services can influence the way in which subjects perceive and evaluate their QoL^22^. This configuration suggests an influence on the development of ways of thinking about QoL because of possible modulation of the possibilities of having access to different resources and fonts of information. 

The self-assessment of health in PLWHA shows the economic class strongly associated with health status: the higher the classification, the better the self-assessment^22^. Thus, the need to increase the economic independence of PLWHA by improving the educational level and inclusion in the formal labor market is evident, helping to generate more income and less insecurity.

Kardecist/Spiritist participants better assessed QoL in the domain that deals with issues related to dependence on ART, as well as the treatment itself. It can be said that spirituality is closely related to the improvement of QoL in patients with chronic diseases^23^. This constitutes an important factor in the perception of QoL, however, it has been neglected in most studies with PLWHA. Nevertheless, it is clear that HIV positive people use their religion/spirituality to cope with the stressors associated with HIV^23^. 

Regarding the temporal analysis in the assessment of QoL, it is thought that the study findings were relevant, as the value of the significance level of the statistical test was shown to be borderline for the cutoff point adopted, which authors attribute, in some cases, to intervening aspects such as the size of the sample used. It is believed that the results found, therefore, deserve consideration and should be further scrutinized, taking into account a previous study regarding this variable^24^.

Thus, regarding the configuration observed in the data for this variable, it is thought that, possibly, PLWHA with a greater length of experience with the diagnosis have established better mechanisms to adapt to their physical condition, with a positive impact on the respective QoL domain. Significant associations among some domains, with worse QoL assessments, could be explained by the fact that the subjects are still adapting to the new condition or have become targets for prejudice and stigmatization of the disease, with direct effects on the QoL^3^ ^,^ ^15^. However, aspects of the physical health and financial condition of PLWHA are revealed as essential in maintaining good QoL, with the effects of this condition affecting different domains^4^ ^,^ ^12^ ^,^ ^15^. The longer time since diagnosis in conjunction with other variables in the PLWHA group over 50 years of age, investigated by the Federal University of São Paulo, were identified as the variables that were most responsible for changes in QoL^25^.

It is therefore suggested, from the findings, the understanding that the treatment of PLWHA in its comprehensive dimension, associated with the greater experience with the diagnosis, anchored in networks of social and spiritual support, is a positive combination reflected in the best perception of QoL in this group.

This study presents limitations, such as the fact that the participants were recruited from a reference service that tends to present samples of people with better living and health conditions, with the consequent overestimation of the QoL scores. On the other hand, this also allowed at least part of the outcome of the healthcare process developed in a unit specialized in HIV/AIDS to be touched upon. The results cannot be generalized to other populations and regions, however, they provide an insight into QoL and its relationship with socioeconomic, health and temporal aspects in PLWHA in the reality of internalization of the epidemic in the Brazilian population.

## Conclusion 

The methodological approach adopted allowed the perception of QoL of the PLWHA, in all domains, to be identified and classified as intermediate. This finding shows that, despite the existence of policies for access to ART and to healthcare available in the public network, other aspects, such as social characteristics, should be considered for the populations in which HIV has spread.

Therefore, the use of the specific instrument for assessing QoL among PLWHA is highlighted as an initiative that remains innovative in a context of internalization. Furthermore, it can provide useful information to support health policies in the prevention, treatment and nursing interventions, as statistical associations were observed regarding the variables gender; employment status; family income; personal income; religious orientation and time since diagnosis, confirming the relevance of the social elements for the needs of different segments of people living with the disease. 

The domains of spirituality and social relationships presented the best evaluations of perception of QoL, constituting essential subsidies in the development of the structures of coping with the condition. Regarding the hypothesis of temporal interference in the assessment of QoL, it is considered that the condition of living with HIV positivity over time represents a reworking of the life processes, particularly those that condition the adjustments and coping in the face of the physical changes brought about by the disease. 

It is thought that maintaining good mental health, together with a network of psychosocial and spiritual support, in addition to receiving the appropriate treatment, from the moment of discovery of the diagnosis, contribute markedly to the better perception of QoL of PLWHA. In view of these considerations, the comprehension of this dynamic by nurses can contribute to their actions, especially care, therapeutic and educational actions, with greater relevance to the life and quotidian of PLWHA, reflecting in improvements in the QoL.
